# Biomechanical evaluation of lumbar spondylolysis repair with various fixation options: A finite element analysis

**DOI:** 10.3389/fbioe.2022.1024159

**Published:** 2022-10-21

**Authors:** Yuchen Ye, Shichang Jin, Yang Zou, Yuekun Fang, Panpan Xu, Zhili Zhang, Nan Wu, Changchun Zhang

**Affiliations:** ^1^ Department of Orthopaedics, First Affiliated Hospital, Bengbu Medical College, Bengbu, China; ^2^ Anhui Province Key Laboratory of Tissue Transplantation, Bengbu Medical College, Bengbu, China

**Keywords:** lumbar spondylolysis, finite element analysis, biomechanical, surgical therapy, internal fixation

## Abstract

**Objective:** This study was designed to compare the biomechanical properties of lumbar spondylolysis repairs using different fixation methods by using three-dimensional finite element analysis.

**Methods:** Five finite element models (A, B, C, D, and E) of L4-S1 vertebral body were reconstructed by CT images of a male patient (A: intact model; B: spondylolysis model; C: spondylolysis model with intrasegmental direct fixation by Buck screw; D: spondylolysis model with intersegmental indirect fixation by pedicle screw system; E: spondylolysis model with hybrid internal fixation). L5-S1 level was defined as the operative level. After the intact model was verified, six physiological motion states were simulated by applying 500 N concentrated force and 10 Nm torque on the upper surface of L4. The biomechanical properties of the three different internal fixation methods were evaluated by comparing the range of motion (ROM), maximum stress, and maximum displacement.

**Results:** Compared with Model B, the ROM and maximum displacement of Model C, D, and E decreased. The maximum stress on L5/S1 disc in models A, B, and C was much higher than that in Model D and E under extension and lateral bending conditions. Under axial rotation and lateral bending conditions, the maximum stress of interarticular muscle and internal fixation system in Model B and Model C was significantly higher than that in Model D and Model E. In contrast to Model D, the stress in Model E was distributed in two internal fixation systems.

**Conclusion:** In several mechanical comparisons, hybrid fixation had better biomechanical properties than other fixation methods. The experimental results show that hybrid fixation can stabilize the isthmus and reduce intervertebral disc stress, which making it the preferred treatment for lumbar spondylolysis.

## Introduction

Lumbar spondylolysis refers to bone discontinuity or bone defect in the transition area of the upper and lower articular and transverse processes of the lumbar spine, and it is one of the important causes of low back pain in youth (Leone et al., 2011; Gagnet et al., 2018). The incidence of lumbar spondylolysis is around 6%–8% in the general population, but it can reach 63% in people who engage in certain physical activities (Sairyo et al., 2005; Goetzinger et al., 2020). The pathogenesis of lumbar spondylolysis is still controversial. However, the most likely mechanism is that stress fractures can occur under the presence of high intensity and frequency of lumbar activity in the congenital weak or dysplastic anatomic weak area of the vertebral spondylolysis (Terai et al., 2010). Based on previously reported cases, lumbar spondylolysis is more common in L4 and L5 vertebrae, as much as 80% of spondylolysis fractures occurred in L5, and may be associated with varying degrees of spondylolisthesis (Berger and Doyle, 2019).

Presently, nonsurgical treatment is still the main treatment for young people with spondylolysis (Goetzinger et al., 2020). However, surgical treatment is required for patients with refractory low back pain or a poor response to nonoperative treatment (Bouras and Korovessis, 2015; Berger and Doyle, 2019). According to previous reports, many techniques for isthmus repair have been described, including the Scott wiring technique, hook-wire constructs, the translaminar interfragmentary screw, and pedicle screw hook structure with bone graft (Yamamoto et al., 2008; Berjano et al., 2020; Kumar et al., 2021; Tarpada et al., 2021).

Currently, there are two common methods of internal fixation in clinical practice. One is Buck’s technique, proposed by Buck in 1970, to directly fix the isthmus repair, (JE, 1970; Snyder et al., 2014)^,^ and the other is the indirect fixation of the isthmus with the intersegmental pedicle screw internal fixation system used by various scholars (Huang et al., 2019; Zhang et al., 2021). Extensive studies have confirmed the effectiveness and safety of both methods. Buck’s technique also conserves segmental motion, allowing for rapid postoperative recovery and minimal blood loss (Giudici et al., 2011). Another pedicle screw system ameliorated the low postoperative fusion rate. The strong grip of the pedicle screw itself and the squeezing effect of the screw system on the isthmic bone graft ensure isthmic fusion (Huang et al., 2019; Zhang et al., 2021). However, the above two fixation methods also have certain defects. For example, although the Buck technique can fix the isthmus directly, it cannot solve the problem of stress concentration in the lumbosacral region (Sairyo et al., 2006a; Sterba et al., 2018; Haj-Ali et al., 2019). On the other hand, the pedicle screw technique can disperse stress from the lumbosacral region, but it cannot ensure isthmus stability. In order to solve the problem of stress concentration in the lumbosacral region. It can also disperse stress from the lumbosacral region to ensure the stability of the isthmus. Therefore, the author also proposed a hybrid fixation method and added it to comparing fixation methods for spondylolysis repair.

Finite element analysis has successfully been used in spine biomechanics research, with the development of computer science (Chosa et al., 2004; Wang et al., 2006). Ramakrishna et al. used finite element analysis to demonstrate the role of sacral slope in the progression of bilateral isthmus defects to lumbar spondylolisthesis in L5 (Ramakrishna et al., 2017). Marwan et al. analyzed the stress distribution of the isthmus, pedicle, and intervertebral disc in a lumbar finite element model by CT reconstruction of a patient with spondylolysis (El-Rich et al., 2006). Sairyo et al. evaluated the biomechanical properties of the Buck technique in repairing lumbar spondylolysis using the finite element method (Sairyo et al., 2006b). Matsukawa et al. compared the biomechanical stability of cortical bone trajectory screws and pedicle screws for isthmus repair (Matsukawa et al., 2016).

**FIGURE 1:**
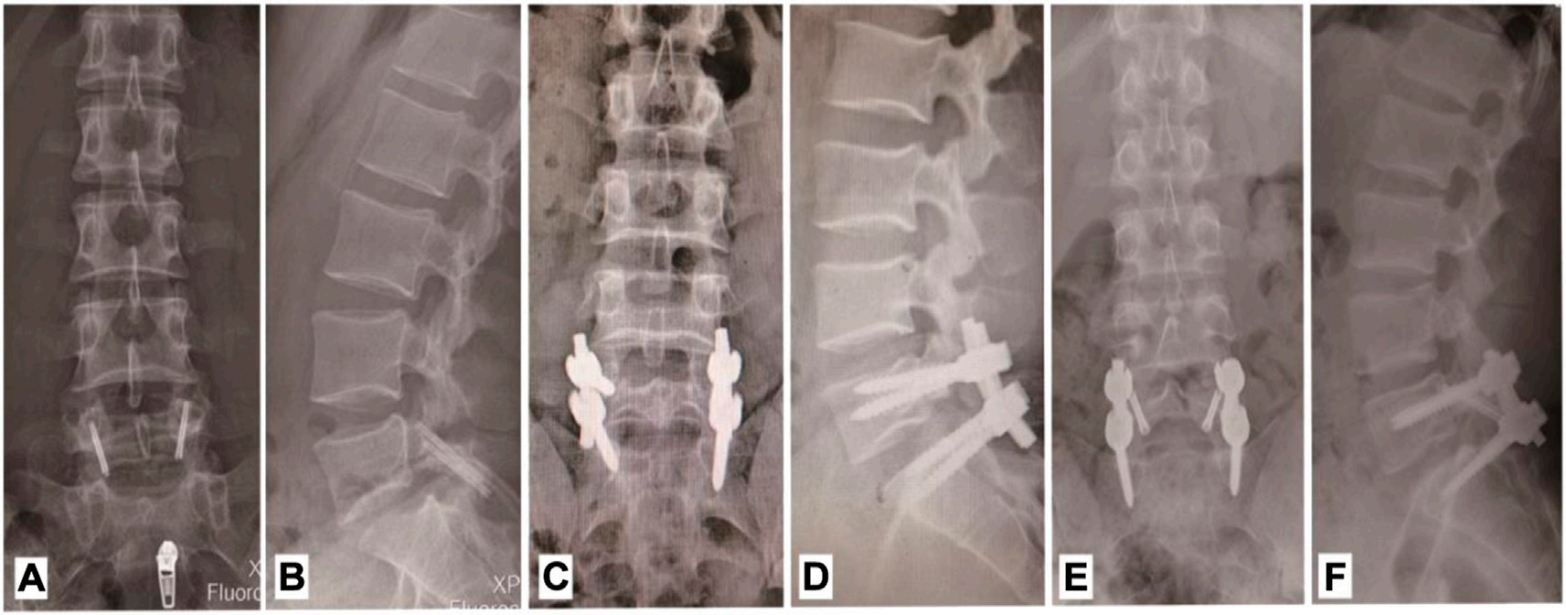
X-ray images of three internal fixation options. **(A,B)** Anteroposterial-lateral radiograph with intra-segmental direct fixation. **(C,D)** Anteroposterial-lateral radiograph with intra-segmental direct fixation. **(E,F)** Anteroposterial-lateral radiograph with hybrid fixation.

In the past, most scholars focused on the finite element study of lumbar spondylolysis with intrasegmental fixation methods (Li et al., 2022a), whereas the comparative study with intersegmental fixation was lacking. In this study, we conducted biomechanical evaluation and comparison of the three surgical methods (Figure 1), including intrasegmental direct fixation, intersegmental indirect fixation, and hybrid fixation, through finite element analysis to offer a theoretical basis for the surgical treatment of young lumbar spondylolysis.

## Materials and methods

### Construction of the finite element model

This study of the original CT data from a 20-year-old patient diagnosed with L5 bilateral spondylolysis (Supplementary Figure S1); the scan slice thickness was 0.6 mm. All 481 CT images were transmitted to Mimics (Mimics 20.0, Materialize, Leuven, Belgium) in DICOM, a medical image processing software, and adjust the appropriate gray scale to obtain a clear bone profile (Wan and Higgins, 2003). Following the completion of the mask processing, the files were exported to STL format. These STL files were then imported into Geomagic Studio 12 (Geomagic, United States) software to reconstruct solid surfaces by inversion. Subsequently, Solid Works software (Dassault Systems, United States) was used to assemble internal fixation and fabricate the disc, articular cartilage, and bone graft. The intervertebral disc was reconstructed according to vertebral anatomy, and the isthmic defect was filled with bone graft. Then Hypermesh software (Altair Technologies, Fremont, CA, United States) was used to mesh solid models of bone and ligament structures. Finally, the finite element analysis software Abaqus 6.10 (Dassault Systemes, France) was used to add soft tissues, such as ligaments, set control conditions, and submit computational solutions.

**FIGURE 2:**
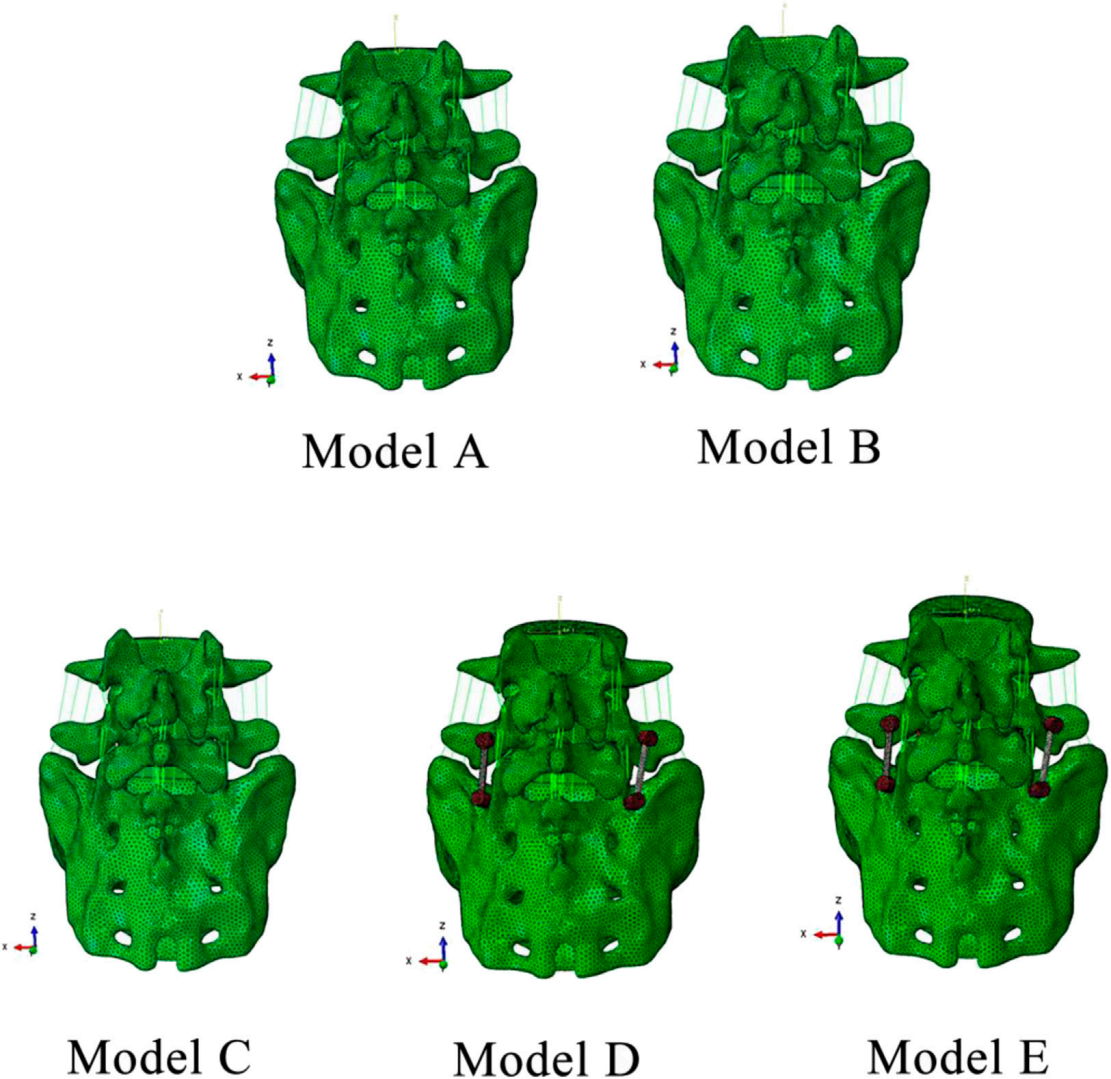
Five finite element models. Model A: Intact model; Model B: Spondylolysis model; Model C: Spondylolysis model with Buck screw; Model D: Spondylolysis model with pedicle screw fixation system; Model E: Spondylolysis model with hybrid fixation.

There are five finite element models established in this experiment (Figure 2). Model A: Intact L4-S1 vertebral model with normal L5 vertebral body after repair; Model B: Original model of CT scan showed bilateral spondylolysis of L5 without internal fixation; Model C: Intrasegmental internal fixation model, in which Buck screws were placed axially on the L5 pars interarticulars of Model B; Model D: Intersegmental internal fixation model, in which L5 and S1 of Model B placing the pedicle screw fixation system; Model E: Hybrid internal fixation model, in which Buck screw and intersegmental pedicle screw fixation system were placed simultaneously on Model B. The operation of the internal fixation model was simulated, and its mechanical properties were analyzed. The model provides a real surgery, said and make the analysis of the mechanical properties are possible.

### Material properties

The finite element model included L4-S1 vertebral body, facet joints, intervertebral disc, ligament system, and internal fixation device. According to the CT image grayscale and bone density, cortical bone and cancellous bone of the vertebral body were assigned different material properties (Baca and Horak, 2007; Song et al., 2021). The intervertebral disc consists of the nucleus pulposus, annulus fibrosus, and upper and lower endplates, which are assigned different material properties. For the model with an internal fixation system, bone grafts were added and assigned using autogenous cancellous bone material properties. The ligament system includes the anterior longitudinal ligament, posterior longitudinal ligament, ligamentum flavum, interspinous ligament, supraspinal ligament, saccular ligament, and transverse ligament. They are configured as homogeneous orthotropic linear elastic materials by obtaining data from references (Baca and Horak, 2007; Song et al., 2021). The detailed materials and properties of the components are illustrated in Table 1.

**TABLE 1:** Material properties of finite element models (Elmasry et al., 2018; Li et al., 2022a).

Structure	Young’s modulus (MPa)	Poisson’s ratio
Cortical bone	12000	0.3
Cancellous bone	100	0.2
Cartilaginous endplate	1200	0.29
Posterior structure	3500	0.25
Annulus fibrosus	6	0.45
Nucleus pulposus	1	0.49
Anterior longitudinal ligament	20	0.3
Posterior longitudinal ligament	20	0.3
Supraspinous ligament	15	0.3
Interspinous ligament	11.6	0.3
Ligamentum flavum	19.5	0.3
Capsular ligament	32.9	0.3
Intertransverse ligament	58.7	0.3
Internal fixation (titanium alloy)	110000	0.3

### Mesh generation

To mesh various solid models, the type of model includes the tetrahedral element, pentahedral element (transition element), shell element (cortical bone), and nonlinear truss element (ligament, only in tension and not in compression). The global element size of the model is 1 mm (determined by model validity verification), and local encryption is carried out in the concerned part to ensure calculation accuracy and speed. The tetrahedral element adopts C3D4 element, the pentahedral element adopts the C3D6 element type, the shell element adopts S4R element type, and the truss element adopts T3D2 element (Li et al., 2022a).

### Loading and boundary conditions

A uniformly distributed 500 N vertical downward concentrated force was applied on the upper surface of L4 to close to physiological state. Then, a torsional moment of 10 nm was imposed to simulate the six spinal physiological activities of flexion, extension, left axial rotation (LAR), right axial rotation (RAR), left lateral bending (LLB), and right lateral bending (RLB). ROM of segment L5-S1 was recorded and compared with adjacent segments. The maximum displacement, intervertebral disc stress, facet stress, internal fixation stress, and stress distribution were compared under various models.

### Indirect validity verification

Indirect validation studies aim to extensively evaluate the reliability of models created using automated algorithms by contrasting results from multiple models (i.e., multiple samples) with experimental data in the literature (Campbell et al., 2016). In this study, we reconstructed the completed model using the original model and then compared the generated complete model with the results of other lumbar FE models in the literature.

## Results

### Mesh generation results

In this study, L4-S1 lumbar spine model was reconstructed, and three different internal fixation systems were analyzed using finite element methods. The basic benchmarking model has 595,295, 181,448 elements of a pedicle screw rod system, 59,708 elements of a simple small screw, 79,958 elements of a screw system, and 64 elements of a truss unit.

### Indirect validation results

**FIGURE 3:**
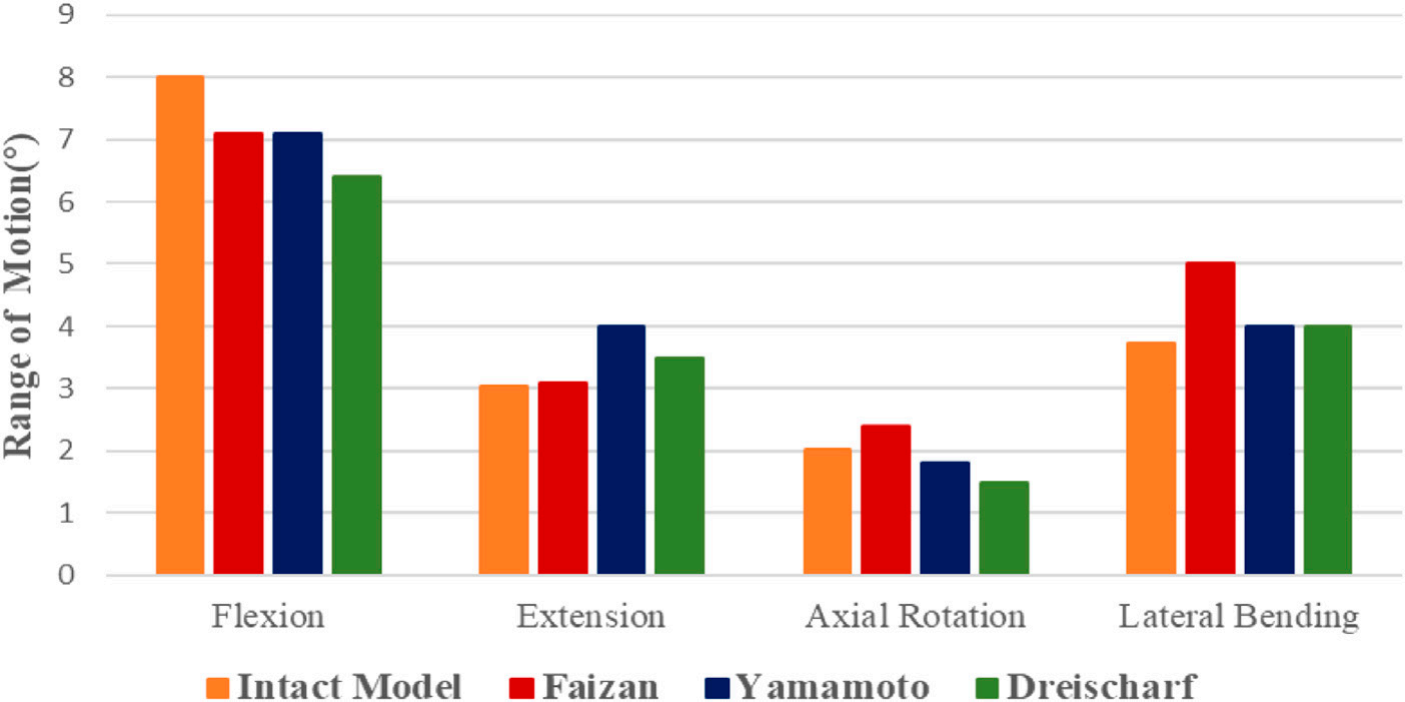
The ROM of the intact model was compared with other studies.

We used the intact model (Model A) as a benchmark against other previously published validation studies (Yamamoto et al., 1989; Dreischarf et al., 2014; Faizan et al., 2014). ROMs of the model subjected to a concentrated force of 500 N and a torque of 10 Nm under four states of motion were compared: bending, stretching, lateral bending, and axial rotation. The load conditions of the model referred to are basically consistent with Model A (Figure 3). The results show that ROM of Model A and other scholar’s research results are basically consistent, indirect proved the validity of the proposed research model.

### Range of motion

**FIGURE 4:**
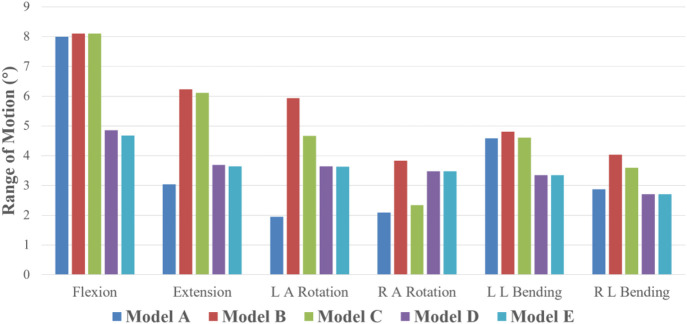
ROM of five finite element models under six motion states.

In all six motion modes, the ROM of model B increased compared with the intact model, with an increase range of 1.36%–205.50%. As displayed in Figure 4, compared with the complete model, the ROM of Model B increased in all six motion modes, with an increasing range of 1.36%–205.50%. Among them, the activity increased significantly under left axis rotation and extension, which were 3.99° and 3.20°, respectively. In addition, in Model B, the activities of left and right axis rotation were 5.93° and 3.84°, respectively, and the activities of left and right lateral flexion were 4.812° and 4.04°, respectively, with noticeable differences, which may be related to the asymmetry of bilateral pedicle spondylolysis in this patient. Compared with Model B, ROM of the three internal fixation models (Model C, D, and E) decreased in all five motion modes except flexion. Among them, the decline range of Model C is 2.02%–38.93%, that of Model D is 9.35%–40.79%, and that of Model E is 9.38%–41.56%. In the comparison of the three internal fixation models, ROM value of Model C decreases the most in the right axis rotation state, while ROM value of Model D and E decreases the most in the extension state, which may be associated to the method of intersegmental internal fixation.

### Maximum displacement of model

**FIGURE 5:**
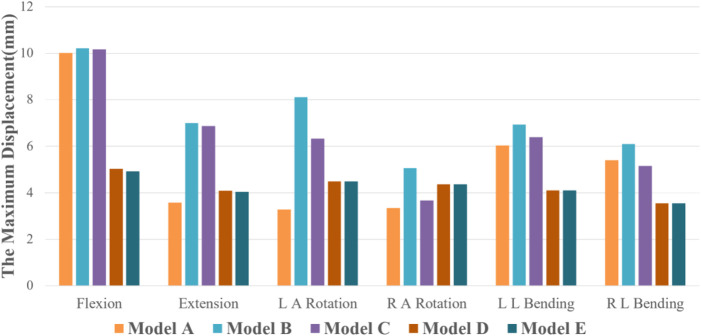
The maximum displacement of five finite element models under six motion states.

As displayed in Figure 5, compared with Model A, the maximum displacement of Model B under six motion states increases with the exceeded range of 1.99%–147.20%, and the displacement difference is the largest under axial rotation. At the same time, the maximum displacement of Model C, D, and E decreased compared with model B under the six motion states. Model C, D, and E decreased by 0.39%–22.06%, 13.78%–50.68%, and 13.86%–51.78%, respectively. The displacement difference between model C and model B is mainly reflected in the axial rotation state, while the displacement difference between Model D and E is significant, except for the axial rotation state on the right side. In addition, the displacement values of Model D and E were not significantly different from those of Model A, except in the flexion state.

Regarding the pars interarticularis (Supplementary Figure S2), the maximum displacement values of Model B and C under the six motion states were all close. Simultaneously, Model D and E under six motion states is significantly different from that of Model B and C. The maximum isthmus displacement of Model D was 22.47% lower than that of Model E in the flexion state, but there was no significant difference between the two models in the other five motion states (extension, lateral bending, axial rotation).

### Maximum von Mises stress of the intervertebral disc

**FIGURE 6:**
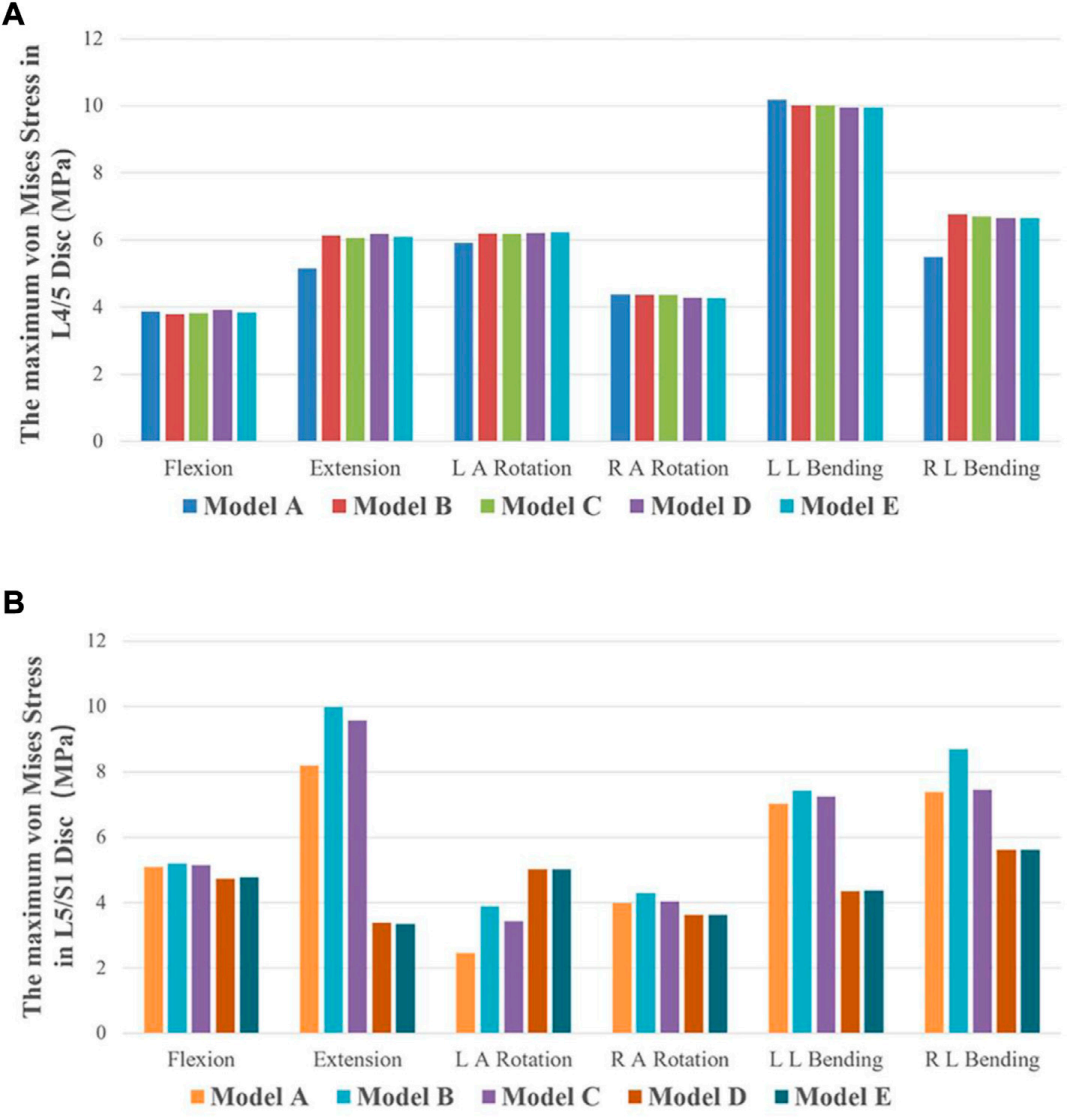
The maximum von Mises stress of discs in five finite element models under six motion states. **(A)** The maximum von Mises stress of L4/L5 IVD in the five models. **(B)** The maximum von Mises stress of L5/S1 IVD in the five models.

The disc stress analysis of the five models showed that the anterior and posterior edges of the disc were concentrated under flexion and posterior extension condition, respectively. In both left and right axial rotation conditions, the stress is concentrated in the anterior and lateral disc. In the left and right lateral bending, the stress distribution is concentrated in the left and right margins of the disc. The maximum stress values of L4/5 and L5/S1 intervertebral discs under different motion states in our five models were compared. As depicted in Figure 6, no significant difference was found between the maximum von Mises stress of L4/5 intervertebral discs (adjacent intervertebral discs) in Model A, B, C, D, and E under the six motion states. In addition to flexion and RAR, the maximum stress on L5/S1 disc was significantly different among the five models. The stress on L5/S1 disc in Model A, B, and C was observably higher than that in Model D and E under extension and lateral bending conditions. Under the LAR, the stress on L5/S1 disc in Model D and E was slightly higher than that in Model A, B, and C. The maximum stress values of Model A, B, and C in the six motion states are as follows: Model B > Model A > Model C. However, the maximum stress values of Model D and E showed no significant difference under any motion state.

### Maximum stress value and stress distribution of pars interarticularis

**FIGURE 7:**
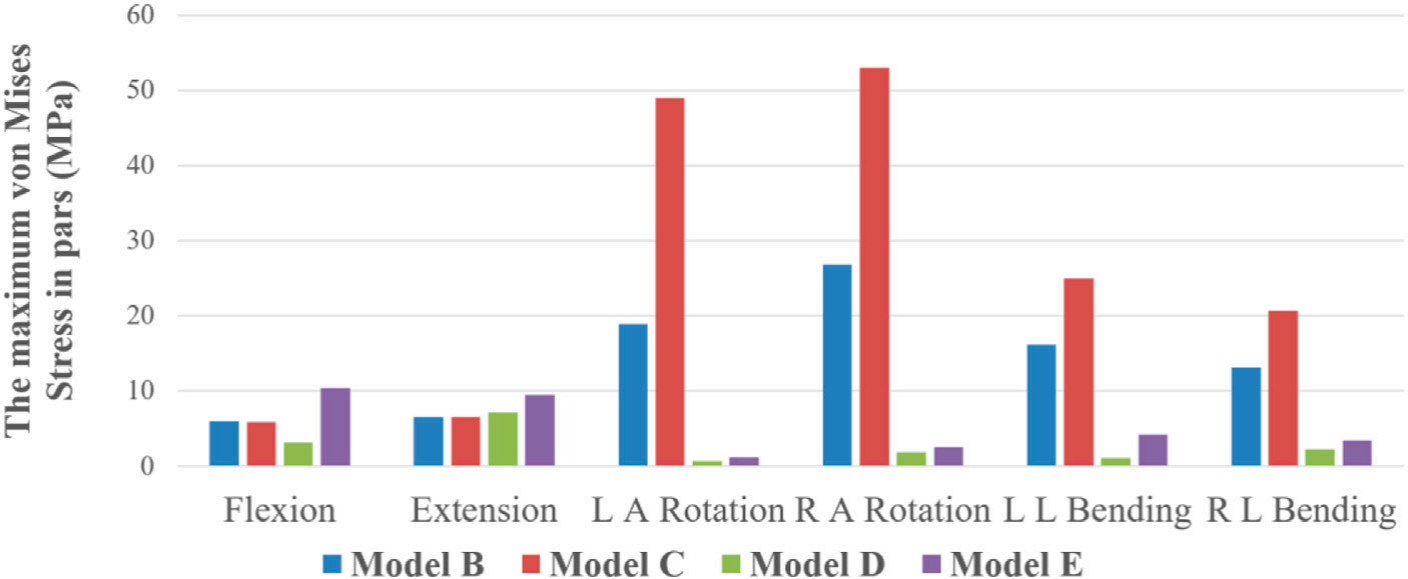
The maximum von Mises Stress on pars interarticularis of Model B, C, D, and E under six states of motion.

**FIGURE 8:**
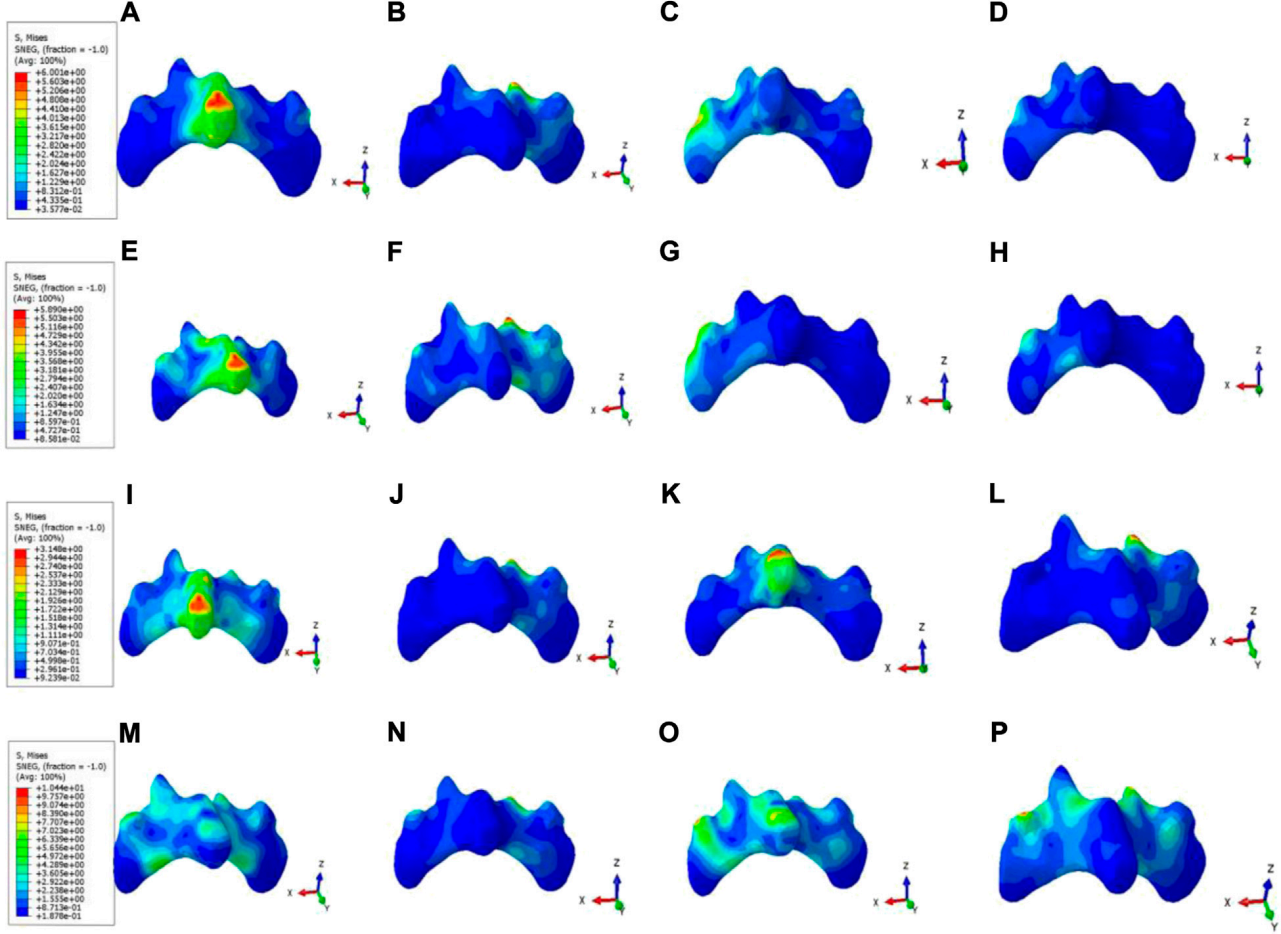
Stress nephograms of pars interarticularis in Model B, C, D, and E under six motion states. **(A–D)** The stress distribution of Model B’s pars interarticularis under flexion, extension, LAR, and LLB, respectively. **(E–H)** The stress distribution of Model C’s pars interarticularis under flexion, extension, LAR, and LLB, respectively. **(I–L)** The stress distribution of Model D’s pars interarticularis under flexion, extension, LAR, and LLB, respectively. **(M–P)** The stress distribution of Model E’s pars interarticularis under flexion, extension, LAR, RAR, LLB, and RLB, respectively.

Model B and C: In the flexion condition, the pars interarticularis stress is concentrated above the spinous process. In the extension and left and right axial rotation conditions, the stress is mainly concentrated on the broken end of the isthmus and below the isthmus. In the left lateral bending and right lateral bending condition, the stress is concentrated on the left and right isthmus fracture end, respectively. Model D: In the flexion condition, the stress is mainly distributed around the spinous process. In the posterior extension, right axial rotation and left and right lateral bending condition, the stress is concentrated on the broken end of the isthmus. Model E: In six different motion states, large stress distribution and no obvious concentration trend. As demonstrated in Figure 7, this difference is negligible in the maximum von Mises stress at the pars interarticularis of Model B, C, D, and E under flexion and extension. However, the maximum von Mises stress sustained by the pars interarticularis in Model B and C was much greater than that in Model D and E under axial rotation and lateral bending conditions. Among them, the maximum von Mises stress in the pars interarticularis of Model C was much greater than that of Model B under axial rotation. Combined with stress nephograms analysis (Figure 8), it can be found that the lateral area of the pars interarticularis of Model B and C were subjected to maximum stress under RAR state, which was 26.87 and 52.98 MPa, respectively. Under the left and right lateral flexion states, the stress of Model B and C was mainly concentrated at the broken end of the isthmus. In addition, compared with Model D, the stress distribution of Model E is more dispersed under the six motion states.

### Maximum stress and stress distribution of internal fixation systems

**FIGURE 9:**
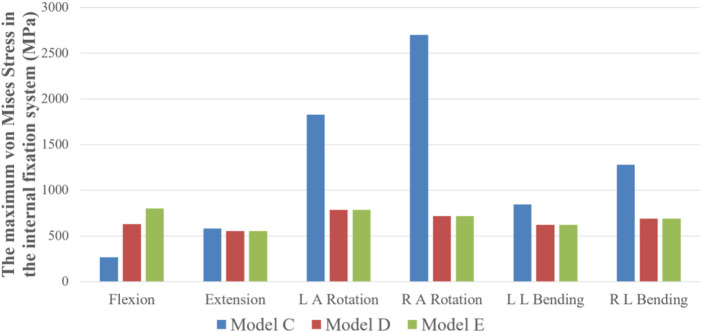
The maximum von Mises stress on the internal fixation system of Model C, D, and E under six states of motion.

**FIGURE 10:**
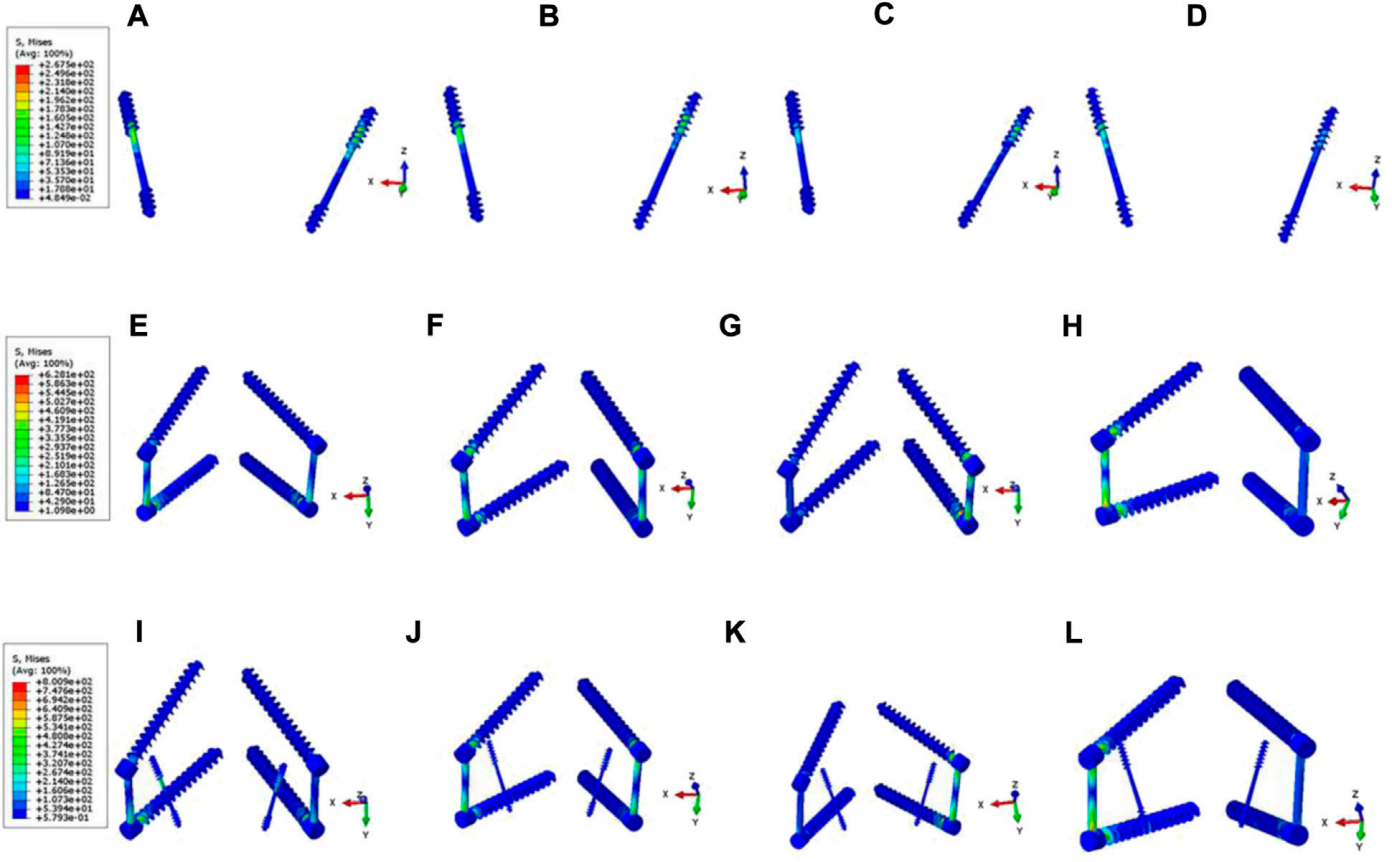
Stress nephograms of internal fixation system in Model C, D, and E under six motion states. **(A–D)** The stress distribution of Model C’s internal fixation system under flexion, extension, LAR, and LLB, respectively. **(E–H)** The stress distribution of Model D’s internal fixation system under flexion, extension, LAR, RAR, LLB, and RLB, respectively. **(I–L)** The stress distribution of Model E’s internal fixation system under flexion, extension, LAR, RAR, LLB, and RLB, respectively.

Model C: In six different motion states, stress of internal fixation occurred at the junction of the left Buck screw and the isthmus defect. Model D: In the flexion and extension condition, the stress mainly focused on the junction of the pedicle screw and the connecting rod. In the left axial rotation and right lateral bending condition, the stress is concentrated at the junction of the right pedicle screw and the connecting rod. In the right axial rotation and left lateral bending condition, the stress is concentrated at the junction of the left pedicle screw and the connecting rod. Model E: During the flexion condition, the stress distribution at the junction of the Buck screw to the isthmus defect and between the pedicle screw and the connecting rod. In the extension condition, the stress is distributed between the pedicle screw and the connecting rod. In the left axial rotation and right lateral bending condition, the stress is concentrated at the junction of the right pedicle screw and the connecting rod. In the right axial rotation and left lateral bending condition, the stress is concentrated at the junction of the left pedicle screw and the connecting rod. As revealed in Figure 9, the maximum von Mises stress of internal fixation in model C was significantly higher than that in Model D and E, except for flexion and extension, and most of the stress was concentrated at the junction of the isthmus defect and screw. Among these, the maximum stress was 2701 MPa, which occurred at the junction between the left Buck screw and the isthmus defect under the RAR state. However, the maximum stress values of Model D and E had no significant difference in the other five motion states except for the flexion state, and the stress distribution was slightly different (Figure 10). Compared with Model D, the stress in Model E was distributed in two internal fixation systems of the Buck screw and pedicle screw under flexion and extension, and the stress area was relatively dispersed. Simultaneously, compared with Model C, the stress distribution of Model E under axial rotation and lateral flexion motion mainly focused on the junction between the pedicle screw and the connecting rod, which greatly reduced the shear force of the isthmus fracture end on the Buck screw.

## Discussion

Based on the underlying pathological mechanism of lumbar spondylolysis and previous literature reports, the key to surgical repair is to fully remove the scar tissue at the broken end of the isthmus and fill it with bone graft, complemented with strong internal fixation, and restore the continuity and integrity of the bone while ensuring the relative stability of the pars interarticularis (Wiltse and Jackson, 1975; Cheung et al., 2006; Mohammed et al., 2018). Therefore, the choice of internal fixation has become the focus of debate among scholars. At present, the mainstream isthmus repair operation is mainly divided into two categories: direct intrasegmental repair, represented by the Buck technique, and indirect intersegmental repair, represented by the pedicle screw technique. According to direct repair, direct compression and fixation of the broken end of the isthmus within the segment can reduce the trauma and preserve the original motion segment (Mohammed et al., 2018). Meanwhile, the concept of indirect repair suggests that intersegmental fixation can limit the ROM of responsible segments in the initial stage of isthmus bone grafting repair, ensure relative stability, and eliminate part of the stress from the lumbosacral region, creating a suitable biomechanical condition for bone fusion in the isthmus (Sterba et al., 2018).

Several researchers have previously analyzed the advantages and disadvantages of various internal fixation methods for the biomechanical evaluation of isthmus repair using computer simulation and finite element algorithm. For example, using the finite element method, Sairyo et al. examined the impact of Buck direct repair on disc stress (Sairyo et al., 2006b). Li et al. conducted a three-dimensional finite element analysis to compare the biomechanical effects of the pedicle screw U-rod internal fixation system and the pedicle screw lamina hook internal fixation system in the treatment of lumbar spondylolysis (Li et al., 2022a). These studies were based on a vertical comparison of the therapeutic effects of different types of internal fixation in intrasegmental repair. This study compared the biomechanical properties of direct intrasegmental and indirect intersegmental repair based on Buck screw fixation and pedicle screw fixation systems. In addition, we designed a new surgical strategy for comparison, aiming to explore whether the hybrid procedure can combine the advantages of the two mainstream procedures while making up for the shortcomings of a single procedure.

This study set up five kind of three-dimensional finite element model based on CT images. First, we use the Model A and Model B between intact model and spondylolysis model to compare the biomechanical differences we compared with Model A, the ROM and maximum displacement of Model B increased under six motion states, especially under extension and axial rotation states. This also suggests that lumbar spondylolysis can lead to decreased spine stability, and long-term instability can lead to vertebral spondylolisthesis. Moreover, combined with the results of intervertebral disc stress analysis, it can be concluded that changes in activity can lead to the biomechanical imbalance of relevant segments, instantaneous rotation center deviation, increased intervertebral disc tissue stress, and risk of degeneration (Sairyo et al., 2006c; Haj-Ali et al., 2019). Compared with Model B, the ROM and maximum displacement of Model C, D, and E decreased due to the addition of an internal fixation device. Among them, Model D and E have smaller ROM and displacement values than Model C, proving that intersegmental fixation can provide better stability in the early postoperative period than intrasegmental fixation. Furthermore, the pedicle screw system can effectively share the stress of the intervertebral disc in a state of spondylolysis due to the supporting role between the segments.

To further compare the stress of the L5 pars interarticularis in different models, we analyzed the maximum displacement and stress nephograms. The results demonstrated that compared with Model B and C, Model D and E with the pedicle screw system for intersegmental fixation significantly reduced the absolute displacement of the pars interarticularis. The results of the stress nephograms depicted that the stress on the pars interarticularis of model C was substantially higher than that of Model B under axial rotation and lateral bending, which might be attributed to the extra binding force of the Buck screw on the pars interarticularis under these conditions. Meanwhile, due to the limitation of intersegmental fixation on the activity of corresponding segments, the maximum displacement and stress of the L5 isthmus in Model D and E were not significantly different. However, due to the existence of multiple internal fixations, the stress distribution on the isthmus defect in Model E was more dispersed than in Model D. This dispersed stress may indicate that there is less shear force in the isthmus of Model E, which is more conducive to the formation of fibrous callus in the early postoperative stage and the shaping of callus in the later stage (Ishida et al., 2018).

On the other hand, the failure risk of loosening and fracture of postoperative internal fixation depends on the magnitude and distribution of stress (Giudici et al., 2011). The stress nephograms demonstrated that the maximum stress magnitude of three internal fixation models under six motion states appeared in the axial rotation state of Model C, which was 2701 mPa. Based on the analysis of the stress distribution of Model A and B, we believe that the vertebral body exerts strong shear force on the broken end of the isthmus and the directly fixed internal fixation device under the axial rotating state due to bone discontinuity in the isthmus region of the vertebral body. This is also consistent with the results of the isthmus stress distribution. Due to the lack of restriction of intersegmental fixation on relative movement (e.g., axial rotation and lateral bending) between vertebral bodies, the isthmus region under direct intrasegmental fixation is subjected to high shear stress. As the Buck technique was based on a screw that fixed the bone at both ends of the isthmus with a single axis and the lack of an effective connection between the two screws, it reduced the limitation of the relative motion of axial rotation and lateral bending between the vertebral bodies (Fan et al., 2010). Subsequently, such low stability may not be conducive to the osseous fusion between the broken ends of the isthmus, and even internal fixation loosening and fracture may occur, resulting in internal fixation failure and surgical failure (Giudici et al., 2011; Tsai et al., 2022). Therefore, compared with direct intrasegmental fixation, indirect intersegmental fixation can provide greater stability and bear less stress. In addition, by comprehensively analyzing the stress cloud of the isthmus and internal fixations, we believe that the mixed fixation method can present a more dispersed stress distribution than the simple intersegmental fixation method. To sum up, we believe that at the early stage after isthmus repair, indirect intersegmental fixation can provide higher stability and smaller shear force at the broken end than direct intrasegmental fixation. In contrast, hybrid fixation can create a more dispersed stress distribution based on simple intersegmental fixation, reducing the risk of internal fixation failure.

In addition, although intersegmental fixation can provide higher initial stability than intrasegmental fixation, the cost of such stability is the long-term loss of partial movement of moving segments after surgery, resulting in reduced lumbosacral flexibility (Li et al., 2022b). This loss in activity may result in lower quality of life and postoperative satisfaction in young patients. Combined with previous reports, we believe that Buck technology combined with short-term intersegmental pedicle screw fixation can ensure the initial osseous fusion of the isthmus. At the same time, the intersegmental fixation device can be removed surgically as early as possible. ROM of the lumbosacral region can be restored through early functional exercise (Meng et al., 2022). Certainly, the hybrid fixation is recently proposed as a new technology combining intrasegmental fixation and intersegment fixation technology, and the material application cost is slightly higher compared with the other two technologies. Fortunately, the initial clinical applications showed that the costs were all within the expected range.

This study compared the biomechanical effects of three kinds of operation based on computer modeling and finite element analysis. First of all, we have to admit the limitations of finite element research, such as: the computer simulated disc constructed by the final version of the fiber ring, upper and lower cartilage is somewhat different from the disc in the real state. For model validation, an indirect verification method is used, which may need to be combined with cadaver specimens for biomechanical at a stage. In addition, this study only compared the advantages and disadvantages of the three surgical methods through computer simulation, which provided a theoretical basis for clinical treatment; however, it lacked practical research. Whether indirect intersegmental fixation is more effective than direct intrasegmental fixation in correcting sagittal balance in patients with lumbar spondylolysis needs to be confirmed by collecting clinical cases and measuring sagittal parameters (Roussouly et al., 2006; Vialle et al., 2007). Meanwhile, postoperative isthmus repair is a long-term dynamic process, and the biomechanical assessment in this study is only based on a 3D model. Hence it lacks credibility in the time dimension (Sakamaki and Sairyo, 1976; Sterba et al., 2018). In conclusion, the degree of intervertebral disc degeneration and the recovery of sagittal balance are objective evaluation criteria after isthmic repair. In contrast, the subjective feelings, such as the degree of pain relief and the improvement in function, need to be analyzed through further clinical studies.

## Conclusion

This study compared three lumbar spondylolysis repair methods based on finite element analysis from a biomechanical perspective. The results demonstrated that although the direct intrasegmental fixation based on the Buck technique could retain the original ROM of the lumbosacral region to a large extent, the relative stability was poor, and the intervertebral disc, isthmus region, and internal fixator had to bear large and relatively concentrated stress under axial rotation and lateral bending. Indirect intersegmental fixation based on pedicle screw technology can provide some initial stability, but stress can become excessively concentrated on the screw and rod joints. The hybrid fixation technique can effectively disperse the stress distribution of the intervertebral disc, isthmus region, and internal fixator under various motion states while ensuring initial phase stability. In conclusion, we believe that hybrid fixation can effectively reduce the risk of internal fixation failure and disc degeneration and provide early stability for bone fusion. Simultaneously, to avoid the loss of lumbosacral flexibility due to intersegmental fixation, we recommend using “temporary” fixation to provide individualized surgical strategies for young patients with lumbar spondylolysis.

## Data Availability

The original contributions presented in the study are included in the article/Supplementary Material, further inquiries can be directed to the corresponding author.
