# Supplementary material for: Biomechanical evaluation of lumbar spondylolysis repair with various fixation options: A finite element analysis
Source: Front Bioeng Biotechnol. 2022 Oct 21;10:1024159. doi: 10.3389/fbioe.2022.1024159 (PMC9634087; doi:10.3389/fbioe.2022.1024159)

**Supplementary Material**

**Biomechanical Evaluation of Lumbar Spondylolysis Repair with Various Fixation Options: A Finite Element Analysis**

Yuchen Ye^a,b,1^ Shichang Jin^a,1^ Yang Zou ^a^ Yuekun Fang ^b^ Panpan Xu^b^ Zhili Zhang ^a^ Nan Wu ^a^ Changchun Zhang ^a,b^*

**Figure S1.** Imaging findings of a 20-year-old male patient


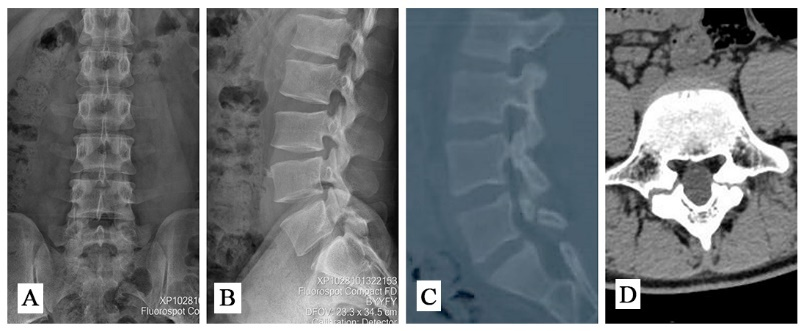


**Figure S2.** The maximum displacement of models B, C, D, and E under six motion states


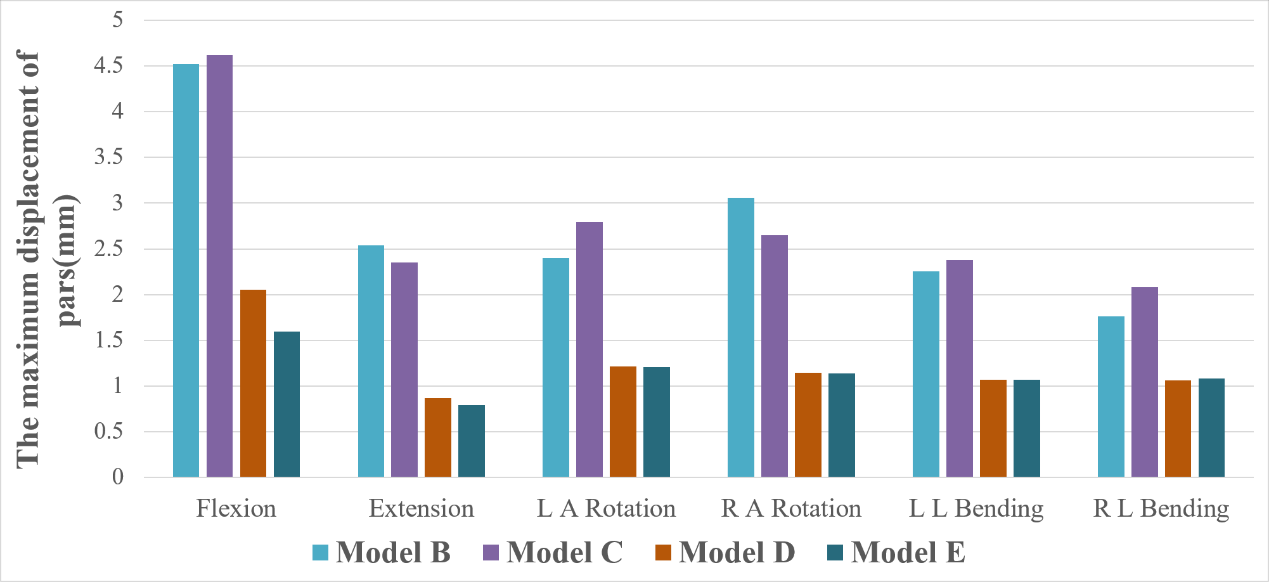

Supplement: Supplementary file 1 [file Table1.DOCX]
